# Human restricted *CHRFAM7A* gene increases brain efficiency

**DOI:** 10.3389/fnins.2024.1359028

**Published:** 2024-04-22

**Authors:** Dejan Jakimovski, Ryu P. Dorn, Megan Del Regno, Alexander Bartnik, Niels Bergsland, Murali Ramanathan, Michael G. Dwyer, Ralph H. B. Benedict, Robert Zivadinov, Kinga Szigeti

**Affiliations:** ^1^Department of Neurology, Buffalo Neuroimaging Analysis Center, Jacobs School of Medicine and Biomedical Sciences, University at Buffalo, State University of New York, Buffalo, NY, United States; ^2^Department of Neurology, Jacobs School of Medicine and Biomedical Sciences, University at Buffalo, State University of New York, Buffalo, NY, United States; ^3^Department of Pharmaceutical Sciences, University at Buffalo, State University of New York, Buffalo, NY, United States; ^4^Center for Biomedical Imaging at the Clinical Translational Science Institute, University at Buffalo, State University of New York, Buffalo, NY, United States

**Keywords:** *CHRFAM7A*, neuropsychological assessment, structural MRI, human brain diversity, efficiency

## Abstract

**Introduction:**

*CHRFAM7A*, a uniquely human fusion gene, has been associated with neuropsychiatric disorders including Alzheimer’s disease, schizophrenia, anxiety, and attention deficit disorder. Understanding the physiological function of *CHRFAM7A* in the human brain is the first step to uncovering its role in disease. CHRFAM7A was identified as a potent modulator of intracellular calcium and an upstream regulator of Rac1 leading to actin cytoskeleton reorganization and a switch from filopodia to lamellipodia implicating a more efficient neuronal structure. We performed a neurocognitive-MRI correlation exploratory study on 46 normal human subjects to explore the effect of *CHRFAM7A* on human brain.

**Methods:**

Dual locus specific genotyping of *CHRFAM7A* was performed on genomic DNA to determine copy number (TaqMan assay) and orientation (capillary sequencing) of the *CHRFAM7A* alleles. As only the direct allele is expressed at the protein level and affects α7 nAChR function, direct allele carriers and non-carriers are compared for neuropsychological and MRI measures. Subjects underwent neuropsychological testing to measure motor (Timed 25-foot walk test, 9-hole peg test), cognitive processing speed (Symbol Digit Modalities Test), Learning and memory (California Verbal Learning Test immediate and delayed recall, Brief Visuospatial Memory Test—Revised immediate and delayed recall) and Beck Depression Inventory—Fast Screen, Fatigue Severity Scale. All subjects underwent MRI scanning on the same 3 T GE scanner using the same protocol. Global and tissue-specific volumes were determined using validated cross-sectional algorithms including FSL’s Structural Image Evaluation, using Normalization, of Atrophy (SIENAX) and FSL’s Integrated Registration and Segmentation Tool (FIRST) on lesion-inpainted images. The cognitive tests were age and years of education-adjusted using analysis of covariance (ANCOVA). Age-adjusted analysis of covariance (ANCOVA) was performed on the MRI data.

**Results:**

*CHRFAM7A* direct allele carrier and non-carrier groups included 33 and 13 individuals, respectively. Demographic variables (age and years of education) were comparable. *CHRFAM7A* direct allele carriers demonstrated an upward shift in cognitive performance including cognitive processing speed, learning and memory, reaching statistical significance in visual immediate recall (FDR corrected *p* = 0.018). The shift in cognitive performance was associated with smaller whole brain volume (uncorrected *p* = 0.046) and lower connectivity by resting state functional MRI in the visual network (FDR corrected *p* = 0.027) accentuating the cognitive findings.

**Conclusion:**

These data suggest that direct allele carriers harbor a more efficient brain consistent with the cellular biology of actin cytoskeleton and synaptic gain of function. Further larger human studies of cognitive measures correlated with MRI and functional imaging are needed to decipher the impact of *CHRFAM7A* on brain function.

## 1 Introduction

*CHRFAM7A,* a uniquely human fusion gene, was recently shown to lead to actin cytoskeleton gain of function leading to a reinforced neuronal matrix with precise connections and enlarged synaptic area due to a shift to lamellipodia dendritic spine. The actin phenotype affects all neuronal substructures, including the cell body, growth cone, and dendritic spine. The reinforced membrane in the presence of *CHRFAM7A* leads to post-developmental adaptation to changes in stiffness associated with biological and pathological processes (Szigeti et al., 2023). The mechanism suggests that CHRFAM7A infers a more efficient and resilient brain.

*CHRFAM7A* is a fusion gene between *CHRNA7*, the subunit of α7 nAChR, and *ULK4* (Sinkus et al., 2015). Based on locus specific dual genotyping 75% of the human population is carrier of the direct, expressed allele (Szigeti et al., 2020). Gene expression localization in the human brain corresponds to areas where α7 nAChR is present (Hellström-Lindahl et al., 1998). α7 nAChR is abundant in the human association cortices and has been associated with cognition (Zoli et al., 2018; Wang et al., 2020; Borroni and Barrantes, 2021). *CHRFAM7A* has two alleles, the direct allele that has been shown to be translated, gets incorporated into the α7 nAChR as up to 3 subunits, and creates a CHRFAM7A/α7 nAChR heteropentamer that is hypomorphic (Benfante et al., 2011; de Lucas-Cerrillo et al., 2011; Wang et al., 2014; Ihnatovych et al., 2019, 2020; Leonard and Benfante, 2023). The inverted allele does not modify α7 nAChR function and its translation remains hypothetical. Data thus far indicate that the inverted allele is non-functional from the α7 nAChR perspective (Sinkus et al., 2015; Szigeti et al., 2020). The direct allele non-carriers are inverted, however the heterozygous individuals in the direct allele carrier group also harbor the inverted allele.

Significant improvements in magnetic resonance imaging (MRI) technology and post-processing techniques today enables reliable quantification of the brain volume and its change throughout the human lifespan. Large databases allow us to define brain volume trajectories that could serve as potent tool toward understanding the healthy aging process and the effect of neurological/psychiatric diseases (Hedman et al., 2012; Bethlehem et al., 2022). Only recently, brain charts that define normative sex-stratified and age-related changes increase the sensitivity in detecting genetic and environmental influences on the brain structure and allow quantification of effect sizes when compared to pathological state (Bethlehem et al., 2022). Moreover, MRI techniques such as functional MRI (fMRI) allow assessment of the functional connectivity, a measure describing the concurrent activity of two brain regions within a network during rest or while performing certain task. These networks constantly adapt throughout the aging process and some modulations such as increased neural efficacy may confer additional resilience to pathology (Dennis and Thompson, 2014; Arenaza-Urquijo and Vemuri, 2018).

One of the most commonly discussed general modifiers of life-long cognitive performance is the cognitive and/or brain reserve (Stern, 2009). The reserve was originally introduced in the field of Alzheimer’s Disease (AD) where patients with greater pre-morbid brain volume and higher educational attainment tent to withstand greater amount of pathology and ultimately have better long-term outcomes (Stern, 2012). The neurocognitive and structural relationship can differ based on multiple modifiable (education, lifestyle and leisure activity) and non-modifiable (genetic background, sex) factors (Levine et al., 2021). The impact of the genetic background on the structural and functional brain function has been previously investigated for well-known dementia risk factors such as *APOE* e4 and BDNF Val66Met polymorphisms (Wisdom et al., 2011; Brown et al., 2014). Investigating other genetic candidates that could potentially influence the neurocognitive-structural MRI correlation would further increase our ability to explain the individual variability throughout the lifespan.

We hypothesize that the translated *CHRFAM7A* allele leads to a neuronal gain of function in the human brain through activation of the actin cytoskeleton. We performed a neurocognitive-structural and resting state functional MRI correlation pilot study on 46 normal human subjects to explore the effect of *CHRFAM7A* on human brain function.

## 2 Materials and methods

### 2.1 Participants

University at Buffalo Institutional Review Board (IRB) approved the study, study ID 030–603069. Informed consent procedure was performed. The healthy participants in this sub-analysis were part of a larger prospective study that aimed at investigating the cardiovascular, environmental and genetic risk factors in multiple sclerosis (CEG-MS study) (Jakimovski et al., 2020). Inclusion criteria included (1) age 18–75, (2) willingness to performed a neuropsychological, MRI and clinical investigation. Exclusion criteria included (1) current status of history of any major neurological or psychiatric diagnosis, (2) use of psychoactive medications, (3) pregnant or nursing mothers, and (4) any contraindications in completing the study procedures (i.e., MRI examination). All participants provided signed consent form.

### 2.2 Motor and cognitive battery

Subjects underwent neuropsychological testing to measure motor, cognitive and psychiatric function. Motor performance was quantified by Timed 25-foot walk test (T25FWT) and 9-hole peg test (9HPT) with dominant and nondominant hand. The study utilized validated neuropsychological assessment with the Brief International Cognitive Assessment for MS (BICAMS) (Langdon et al., 2012) which included cognitive measures of processing speed by the Symbol Digit Modalities Test (SDMT), verbal learning and memory (California Verbal Learning Test – 2nd Version, investigating the immediate and delayed recall; CVLT-II) and visual learning and memory (Brief Visuospatial Memory Test – Revised; BVMT-R, investigating immediate and delayed recall) were administered by a trained psychometrist under supervision of licensed neuropsychologist (RHB). In all aforementioned cognitive measures, higher score indicates better cognitive performance. Depression and fatigue were quantified by the Beck Depression Inventory – Fast Screen (BDI-FS) and the Fatigue Severity Scale (FSS). In both patient-reported outcomes, higher scores indicate worse depressive and fatigue symptoms.

### 2.3 Locus specific dual genotyping

Genomic DNA was isolated from whole blood. *CHRFAM7A copy number by TaqMan assay*: Primers (forward primer: GTAATAG TGTAATACTGTAACTTTAAAATGTGTTACTTGT, reverse primer: AGCCGGGATGGTCTCGAT) and probe (TCCTGACTGTACAC ATAAAA) were designed to detect the breakpoint sequence (Applied Biosystems). The duplex real-time PCR assays were performed using a FAM dye-labeled assay targeted to CHRFAM7A and the VIC dye-labeled RNaseP (TaqMan copy number reference assay, part # 4403326) as a reference gene. Each sample was assayed in quadruplicate by using 10 ng DNA in each reaction. Realtime PCR was performed using the CFX384 Real-time PCR Detection System (Bio-Rad). Threshold cycle (Ct) values were determined for CHRFAM7A and compared with Ct values for RNase P. Relative quantity was determined by the DD Ct method.

*CHRFAM7A 2 bp deletion assay*: Genotyping for the 2 bp deletion polymorphism was by limited cycle fluorescent PCR (21 cycles of 948C/30 s, 588C/30 s, 728C/1.5 min) using primers flanking the 2 bp deletion. 2bpForFAM GGGCATATTCAAGAGTTCCTGCTAC and 2bpRev.

CCACTAGGTCCCATTCTCCATTG gave a product size of 170 bp in the absence of the deletion and 168 bp in its presence. PCR products were resolved using a 3,100 fluorescent genotyper and Genemapper v3.0 software to ascertain 170 bp:168 bp ratios. Each assay was.

performed at least twice.

### 2.4 MRI acquisition

All healthy participants were scanned using the same 3 T GE Signa Excite Scanner (GE, Milwaukee, WI) and 8-channel head and neck coil. The sequences that are relevant for the analysis in this study were: (1) 3D high-resolution T1-weighted inversion recovery fast spoiled gradient echo with echo time (TE) of 2.8 ms, repetition time (TR) of 5.9 ms and inversion time of 900 ms, flip angle of 10 degrees, field-of-view of 25.6 × 19.2 cm^2^ and isotropic 1 × 1 × 1 mm slices, (2) functional MRI (fMRI) that acquired 240 volume of gradient echo-echo planar images with TE of 35 ms, TR of 2,500 ms, flip angle of 90 degrees and 3.75 × 3.75 × 4 mm slices and (3) fluid-attenuated inversion recovery (FLAIR) sequence with TE of 120 ms, TR of 8,500 and TI of 2,100 ms, flip angle of 90 degrees, echo train length 1 × 1 × 3 mm slices with no gap. There were no software changes during the acquisition of all participants.

### 2.5 MRI processing

The T2 lesion volume (LV) was determined on FLAIR scans by experienced neuroimager using semi-automated contouring and thresholding tool and corrected with the Java Image Manipulation software (JIM, Xinapse systems, Essex, UK, version 8.0) (Bergsland et al., 2021). All T1 weighted images were preprocessed for N4 bias field correction and lesion inpainting. The segmentation of the brain volumes including the whole brain volume (WBV), white matter volume (WMV), gray matter volume (GMV), lateral ventricular volume (LVV), deep gray matter volume (DGMV) and thalamic volume were performed using the cross-sectional Structural Image Evaluation, using Normalization, of Atrophy (SIENAX^1^) and FMRIB’s Integrated Registration and Segmentation Tool (FIRST^2^) protocols. All volumes were normalized for the head size. The cortical parcellation was performed using the FreeSurfer protocol that provides the cortical map of 86 regions based on the Desikan-Killiany atlas^3^ (Desikan et al., 2006).

The resting-state fMRI was processed using FSL tools as described elsewhere, following Human Connectome Project preprocessing recommendations (Bartnik et al., 2023). Briefly, the processing included removal of the first 2 volumes, slice timing correction, motion correction, intensity normalization, high-pass temporal filtering (2,000 s), field map unwarping based on phase-reversed acquisitions (blipup/down), and 4-mm spatial smoothing. Motion confounds (of the 6 rigid-body parameter timeseries), cerebrospinal fluid signal, and white matter signal were regressed out (Smith et al., 2013). The activity between regions and their functional connectivity was determined by assessing the concordance of temporal activation using partial correlation estimation using Nilearn (Abraham et al., 2014). Matrices of the 86 × 86 regions and their paired functional connectivity were produced.

Instead of functional analysis of all 7,396 potential pairs of cortical regions, the study focused on 9 pre-determined networks including: (1) auditory network, (2) default mode network, (3) executive control network, (4) left hemisphere frontoparietal network, (5) right hemisphere frontoparietal network, (6) sensory-motor network, and (7) three different visual networks (V1, V2, and V3) (Smith et al., 2009). The interpretation of the networks and their behavioral domain mapping is described in detail elsewhere (Smith et al., 2009). The first visual network corresponds explicitly to visual domains whereas V2 and V3 correspond to cognition-language-orthography and cognition-space paradigms, respectively (Smith et al., 2009). The functional connectivity measures of the aforementioned networks were derived using the Brain Connectivity Toolbox (BCT^4^) (Rubinov and Sporns, 2010). The specific network efficiency (how efficiently information is exchanged) was used selected as one proxy measure of integration that will be compared in this study (Latora and Marchiori, 2001).

### 2.6 Statistical analysis

Power calculation was not performed due to the exploratory nature of this clinical-imaging-genetic correlation study thus sample size was not predetermined. Significance was set at *p* < 0.05. Additional correction for false discovery rate (FDR) using the Benjamini-Hochberg procedure was performed and FDR-corrected *p*-values were also shown. The data distribution was determined by visual inspection of the histograms and Q-Q plots. Age was compared using analysis of variance (ANOVA). The non-parametric data was compared using Mann Whitney U test. Comparison between CHRFAM7A carriers and non-carriers for cognitive data were age and years of education-adjusted using analysis of covariance (ANCOVA) and depicted as estimated marginal means (standard error). MRI measures were compared by age-adjusted analysis of covariance (ANCOVA) and described as mean (standard error) and as estimated marginal means corrected for age. SPSS (Armonk, NY, United States) version 28 statistical software was used for all analyses and GraphPad Prism (San Diego, CA, United States) was used for data visualization and creation of the data plots.

## 3 Results

**Figure 1:**
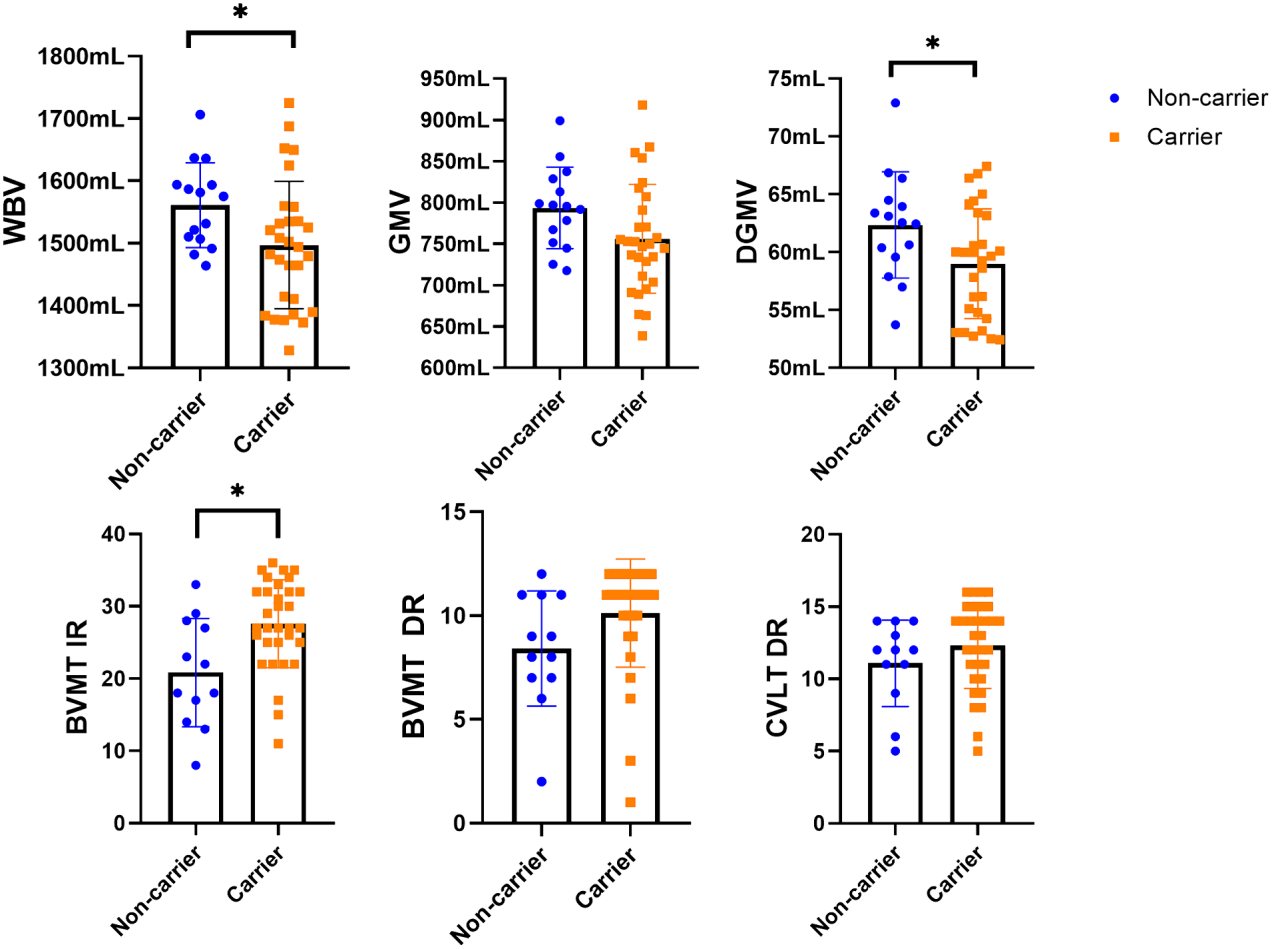
The differences in MRI data between the study groups. LV, lesion volume; WBV, whole brain volume; WMV, white matter volume; GMV, gray matter volume; CV, cortical volume; DGM, deep gray matter volume. *Statistically significant differences (*p* < 0.05).

The study participants had a mean age of 51.8 (SD = 14.9), and 69% were females and 89% white. 71.7% of participants were *CHRFAM7A* direct allele carriers consistent with previously reported frequencies in Caucasians (Benfante et al., 2011; Sinkus et al., 2015; Szigeti et al., 2020; Leonard and Benfante, 2023). Demographic variables (age and years of education) were comparable between direct allele carriers and non-carriers (Table 1). Motor function measured by 25FTW and 9PHT was similar between CHFRAM7A direct allele carriers and non-carriers and depression scores (BDI-FS) were low for both groups (Table 1). Cognitive measures of processing speed (SDMT), verbal and visual learning and memory were consistently higher in the *CHRFAM7A* direct allele carrier-group, reaching statistical significance in visual immediate recall (27.5 vs. 21.1, *p* = 0.003, partial η = 0.202, FDR corrected *p*-value = 0.018) (Table 1, Figure 1). Structural MRI suggested larger whole brain volume in the non-carriers (1549.4 mL vs. 1503.1 mL, uncorrected *p* = 0.046, partial η = 0.094) in the context of similar lateral ventricular volume (38.9 mL vs. 37.0 mL, *p* = 0.708, partial η = 0.03) and T2-lesion volume (0.6 mL vs. 0.6 mL, *p* = 0.876, partial η = 0.001) (Table 2, Figure 1). The smaller WBV in *CHRFAM7A* direct allele carriers was a result of smaller white matter (742.5 mL vs. 764.4, uncorrected *p* = 0.077, partial η = 0.074), gray matter (760.6 mL vs. 785.0 mL, uncorrected *p* = 0.082 partial η = 0.072) and deep gray matter volumes (59.2 mL vs. 61.9 mL, *p* = 0.050 partial η = 0.09). The structural MRI analysis is likely underpowered in this exploratory study (Table 2).

**Table 1:** Differences in physical and cognitive characteristics between the carrier and non-carrier groups.

Physical and cognitive characteristics	Total HC population (*n* = 46)	Direct carrier (*n* = 33)	Direct non-carrier (*n* = 13)	*p*-value	FDR-corrected *p*-value	Partial η
Age, mean (SD)	51.8 (14.9)	50.3 (13.3)	55.5 (18.5)	0.29	–	–
Female/male ratio (F/M)	32/14	24/9	8/5	0.551	–	–
Years of education, mean (SD)	14.6 (2.4)	14.4 (2.3)	15.3 (2.7)	0.319	–	–
T25FWT, median (IQR)	4.5 (3.9–5.0)	4.5 (3.9–4.9)	4.5 (4.3–5.2)	0.409	–	–
9HPT dominant, median (IQR)	20.4 (17.6–22.4)	20.4 (16.9–22.2)	21.0 (17.9–22.9)	0.302	–	–
9HPT non-dominant, median (IQR)	20.6 (18.4–23.4)	20.3 (18.3–23.2)	21.2 (18.5–25.5)	0.458	–	–
9HPT, median (IQR)	20.3 (17.8–23.2)	20.2 (17.8–23.1)	20.8 (18.3–24.2)	0.368	–	–
BDI-FS, median (IQR)	0.0 (0.0–2.0)	1.0 (0.0–2.0)	0.0 (0.0–1.0)	0.367	–	–
FFS, median (IQR)	2.6 (1.8–3.1)	2.6 (1.5–2.8)	3.0 (2.4–4.1)	**0.049**	–	–
SDMT, mean (SD)	55.9 (12.9)	57.4 (1.8)	52.1 (3.0)	0.149	0.179	0.051
CVLT-IR, mean (SD)	54.6 (10.5)	56.1 (1.7)	50.7 (2.7)	0.104	0.156	0.065
CVLT-SR, mean (SD)	12.0 (2.9)	12.2 (0.5)	11.7 (0.8)	0.576	0.576	0.008
CVLT-DR, mean (SD)	11.9 (2.9)	12.4 (0.5)	10.9 (0.7)	0.094	0.188	0.068
BVMT-R IR, mean (SD)	25.8 (7.1)	27.5 (1.0)	21.1 (1.7)	**0.003**	**0.018**	**0.202**
BVMT-R DR, mean (SD)	9.7 (2.7)	10.1 (0.4)	8.5 (0.7)	0.065	0.195	0.082

HC, healthy control; T25FWT, Timed 25-foot walk test; 9HPT, 9-hole peg test; BDI-FS, Beck’s Depression Inventory – Fast Screen; FSS, Fatigue Severity Scale; SDMT, Symbol Digit Modalities Test; CVLT, California Verbal Learning Test; BVMT-R, Brief Visuospatial Memory Test – Revised; IR, immediate recall; SR, short recall; DR, delayed recall; SD, standard deviation; IQR, interquartile range.

The age was compared using analysis of variance (ANOVA). Sex was compared using chi-square test. The non-parametric data was shown as medians (interquartile range) and compared using Mann Whitney U test. The cognitive tests were age and years of education-adjusted using analysis of covariance (ANCOVA). Their results are shown as mean (standard error). *p*-values lower than 0.05 were considered statistically significant and shown in bold.

Additional correction for false discovery rate (FDR) was performed using Benjamini-Hochberg procedure. FDR-adjusted *p*-values were additionally shown in the table.

**Table 2:** Differences in volumetric MRI measures between the carrier and non-carrier groups.

MRI characteristics	Total HC population (*n* = 46)	Direct carrier (*n* = 33)	Direct non-carrier (*n* = 13)	*p*-value	FDR-corrected *p*-value	Partial η
Age	51.8 (14.9)	50.3 (13.3)	55.5 (18.5)	0.29	–	–
T2-LV	0.7 (1.5)	0.6 (0.2)	0.6 (0.3)	0.876	0.876	0.001
WBV	1518.9 (96.2)	1503.1 (13.0)	1549.4 (18.2)	**0.046**	0.368	**0.094**
WMV	749.9 (42.4)	742.5 (7.0)	764.4 (9.8)	*0.077*	0.154	0.074
GMV	768.9 (62.8)	760.6 (7.9)	785 (11.1)	*0.082*	0.131	0.072
CV	624.5 (53.4)	620.3 (6.9)	632.7 (9.6)	0.3	0.4	0.026
LVV	37.6 (16.5)	37.0 (2.6)	38.9 (4.3)	0.708	0.809	0.03
DGMV	60.1 (4.9)	59.2 (0.8)	61.9 (1.1)	**0.05**	0.2	**0.09**
Thalamus	20.2 (1.9)	19.8 (0.3)	20.8 (0.4)	*0.068*	0.181	0.079

LV, lesion volume; WBV, whole brain volume; WMV, white matter volume; GMV, gray matter volume; CV, cortical volume; LVV, lateral ventricular volume; DGMV, deep gray matter volume.

Age-adjusted analysis of covariance (ANCOVA) was performed. All measures are described as mean (standard error) and as estimated means corrected for age. *p*-values lower than 0.05 were considered as statistically significant and shown in bold, whereas *p*-values lower than 0.1 were considered as trends and shown in italic.

Additional correction for false discovery rate (FDR) was performed using Benjamini-Hochberg procedure. FDR-adjusted *p*-values were additionally shown in the table.

Lastly, 37 (80%) out of the 46 participants had an available fMRI sequence as part of their protocol and their functional connectivity was analyzed. In particular, 28 direct allele careers and 9 direct allele non-carriers were included in this comparison (Table 3). In this exploratory fMRI analysis, the *CHRFAM7A* direct allele carriers had lower connectivity within the V2 network resting activity when compared to direct allele non-carriers (FDR corrected p-value = 0.027).

**Table 3:** Differences in the efficiency of the resting-state functional connectivity between the carrier and non-carrier groups.

Rs-fMRI network efficiency	Direct carrier (*n* = 28)	Direct non-carrier (*n* = 9)	*p*-value	FDR-corrected *p*-value
Age, mean (SD)	51.9 (12.6)	53.6 (16.3)	0.746	–
Female/male ratio (F/M)	19/9	7/2	0.321	–
Years of education, mean (SD)	14.32 (2.5)	15.4 (3.0)	0.265	–
Auditory network	0.103 (0.021)	0.113 (0.013)	0.197	0.887
Sensory-motor network	0.128 (0.016)	0.13 (0.019)	0.78	1.000
DMN	0.111 (0.016)	0.115 (0.008)	0.539	0.97
Visual network 1	0.207 (0.045)	0.22 (0.027)	0.432	0.972
Visual network 2	0.158 (0.048)	0.213 (0.036)	**0.003**	**0.027**
Visual network 3	0.113 (0.019)	0.117 (0.023)	0.613	0.919
Executive control network	0.092 (0.013)	0.093 (0.013)	0.867	0.867
Right frontoparietal network	0.113 (0.023)	0.114 (0.027)	0.845	0.95
Left frontoparietal network	0.114 (0.022)	0.107 (0.016)	0.361	1.000

HC, healthy control; SD, standard deviation.

All network efficiencies are shown as mean (standard deviation). Sex was compared using chi-square test. All comparisons were performed using analysis of variance (ANCOVA). *p*-values lower than 0.05 were considered statistically significant and shown in bold.

Additional correction for false discovery rate (FDR) was performed using Benjamini-Hochberg procedure. FDR-adjusted *p*-values were additionally shown in the table.

## 4 Discussion

*CHRFAM7A* is one of the human restricted genes created by fusion between *CHRNA7* and part of *ULK4*. Mechanistic insights from isogenic induced pluripotent stem cell (iPSC) models identified a direct allele mediated actin cytoskeleton gain of function. Consistent with emerging literature that functional effects of the human specific genes are most frequently observed in the brain, immune system and metabolism we demonstrated gain of function in the neuronal lineage and previously have shown immune gain of function in microglia (Ihnatovych et al., 2020; Szigeti et al., 2023). Human specific genes are thought to drive traits that make us human and typically are present in all humans while mutations or absence lead to developmental consequences or disease. *CHRFAM7A* thus far the only human specific gene that is not developmentally essential as 0.7% of the population without *CHRFAM7A* alleles are indistinguishable from carriers, suggesting that it may provide different context for disease when present. Furthermore, the locus is biallelic, harboring direct and inverted *CHRFAM7A*. suggesting that the contextual consideration divides the human population 1–3, or 25% non-carriers and 75% carriers of the direct, translated allele. Early data suggest that allele frequencies differ in racial and ethnic groups perhaps adding an additional layer of diversity for disease context.

Recently we reported that CHRFAM7A translated from the direct allele leads to an actin cytoskeleton gain of function in the neuronal lineage in the form of switching cell membrane structure from filopodia to lamellipodia, and as a result leads to reinforced synaptic structure, adaptation to tissue stiffness and axon guidance. These structural changes suggest a more efficient synapse and we proceeded to test the hypothesis in a neurocognitive-structural and resting state functional MRI correlation study. The study population is very similar to clinical trial populations in neuropsychiatric diseases consisting of mostly Caucasian participants and 2/3 being women. Motor and cognitive measures demonstrated a split as motor function was not affected by *CHRFAM7A* direct allele carrier status while the cognitive measures for processing speed, learning and memory consistently favored carriers, reaching statistical significance on immediate visual learning. The greater differences within the BVMT-R immediate versus delayed tasks suggest that the *CHRFAM7A* direct allele may influence the process of active learning and working memory versus improving the process of consolidation and/or decreasing the memory decay over time. These findings correlate and colocalize with the high expression of the α7 nACHR in the hippocampus and the medial prefrontal cortex which are essential regions for memory processing (Pastor and Medina, 2023). Understanding whether verbal and visual memory are affected differentially requires larger well-powered studies.

BVMT-R requires visual spatial perception in that examinees are awarded points for the accuracy of reproducing what they see, and the location of stimuli. BVMT-R is associated with right hemisphere visual networks in MS (Fuchs et al., 2019). Visual spatial function in middle age has emerged as the main predictor of cognitive decline later in life, and this correlation has been hypothesized as the first measurable decline (Possin, 2010; Salimi et al., 2018; Kolli et al., 2021; Berente et al., 2022). Informed by the underlying cell biology of CHRFAM7A, the association between visual spatial function and cognitive decline could be driven by a more resilient neuronal structure measured by the visual spatial relative strength, latently detecting *CHRFAM7A* direct allele carriers.

Structural MRI validated the prediction for a more efficient neuronal structure from the iPSC model. Larger brain size has been associated with higher cognitive function traditionally, thus the inverse relationship between t whole brain volume and cognitive function in *CHRFAM7A* direct allele carriers accentuates the enhanced cognitive findings. The resting state functional MRI findings further support the more efficient neural structure hypothesis in the sense that direct allele carriers require less connectivity in the unstimulated resting state.

Further studies are needed to validate these findings and to expand how *CHRFAM7A* direct allele affects higher cognitive function through α7 nAChR associated domains such as learning and memory and how it contributes to cognitive reserve. The role of the α7 nAChRs in memory comes mostly from rodent and other animal model experiments and experimental restrictions in humans preclude direct confirmation of the molecular mechanism. Of note, α7 nAChRs agonists, such as encenicline has been studied in clinical trials in both Alzheimer’s disease and schizophrenia and while animal models showed consistent efficacy all human clinical trials failed identifying a translational gap (NCT01969136 and NCT01969123) (Prickaerts et al., 2012). While CHRFAM7A modifies the α7 nAChR into a hypomorphic ionotropic receptor, at the same time shifts Ca^2+^ dynamics in the neuron that activates the actin cytoskeleton (Szigeti et al., 2023). Actin cytoskeleton is a fundamental mechanism in memory formation through stabilizing and maturing synapses resulting in long term potentiation that could account for the observed memory gain of function (Lamprecht, 2021). Synaptic actin dynamics has been shown to contribute to behavioral visual acuity measured by the visual water maze task in rats (Bi et al., 2021). Thus, similar to other human restricted genes (ARHGAP11B, SRGAP2) an apparent loss of function mutation leads to a novel gain of function and the observed outcome is the sum of these effects (Heide et al., 2020).

There are several limitations to our study as it is exploratory in nature. While the sample size and the genotype distribution for carriers and non-carriers were meaningful and consistent with previously reported allele frequencies in Caucasians, we need larger and more diverse studies to confirm these findings. This cross-sectional study does not address whether the efficiency translates into cognitive reserve. Larger longitudinal datasets are needed to answer this very important question. Moreover, we only utilized a small set of tests that only investigated three cognitive domains of cognitive processing speed, verbal and visuospatial learning and memory. Future studies can include greater number of tests that will include measures of executive function, logical memory, visual integration and language. While the inverted allele does not seem to affect α7 nAChR function, it has been implicated in psychiatric disease. Understanding the function of the inverted allele will refine the interpretation of these findings. Due to the small sample size, we restricted the analysis on pre-determined and established networks and did not perform an independent difference in functional connectivity between the direct allele carriers vs. non-carriers. Future more comprehensive fMRI analysis would be able to determine differences that may be more specific to the *CHRFAM7A*.

These preliminary data indicate that human brain may have unique characteristics due to an actin cytoskeleton gain of function in 75% of the population and raises the possibility that this structural change may contribute to resilience and cognitive reserve. Cognitive reserve has been implicated in brain aging and susceptibility to disease thus considering *CHRFAM7A* genotype may facilitate risk stratification and identify new targetable mechanisms for neurodegeneration, neuroinflammation and stroke.

## Data availability statement

The raw data supporting the conclusions of this article will be made available by the authors, without undue reservation.

## Ethics statement

The studies involving humans were approved by University at Buffalo, State University of New York. The studies were conducted in accordance with the local legislation and institutional requirements. The participants provided their written informed consent to participate in this study.

## Author contributions

DJ: Writing – original draft, Writing – review & editing, Data curation, Formal analysis, Investigation, Methodology, Project administration, Visualization. RD: Data curation, Investigation, Methodology, Project administration, Writing – original draft, Writing – review & editing. MRe: Data curation, Investigation, Methodology, Project administration, Writing – review & editing. AB: Data curation, Investigation, Methodology, Project administration, Writing – review & editing. NB: Data curation, Investigation, Methodology, Project administration, Writing – review & editing. MRa: Data curation, Investigation, Methodology, Project administration, Writing – review & editing. MD: Data curation, Investigation, Methodology, Project administration, Writing – review & editing. RB: Data curation, Investigation, Methodology, Project administration, Writing – review & editing. RZ: Data curation, Funding acquisition, Investigation, Methodology, Project administration, Writing – review & editing. KS: Conceptualization, Data curation, Funding acquisition, Investigation, Methodology, Project administration, Resources, Supervision, Writing – original draft, Writing – review & editing.

## Footnotes

^1^ https://fsl.fmrib.ox.ac.uk/fsl/fslwiki/SIENA

^2^https://fsl.fmrib.ox.ac.uk/fsl, version 6.0.

^3^ http://surfer.nmr.mgh.harvard.edu

^4^ http://www.brain-connectivity-toolbox.net/
